# Multi-omics of *in vitro* aortic valve calcification

**DOI:** 10.3389/fcvm.2022.1043165

**Published:** 2022-11-03

**Authors:** Daria Semenova, Arsenii Zabirnyk, Arseniy Lobov, Nadezda Boyarskaya, Olga Kachanova, Vladimir Uspensky, Bozhana Zainullina, Evgeny Denisov, Tatiana Gerashchenko, John-Peder Escobar Kvitting, Mari-Liis Kaljusto, Bernd Thiede, Anna Kostareva, Kåre-Olav Stensløkken, Jarle Vaage, Anna Malashicheva

**Affiliations:** ^1^Institute of Cytology Russian Academy of Science, St. Petersburg, Russia; ^2^Almazov National Medical Research Center Russia, St. Petersburg, Russia; ^3^Heart Physiology Research Group, Division of Physiology, Institute of Basic Medical Sciences, University of Oslo, Oslo, Norway; ^4^Oslo University Hospital, Oslo, Norway; ^5^Centre for Molecular and Cell Technologies, St. Petersburg State University, St. Petersburg, Russia; ^6^Laboratory of Cancer Progression Biology, Cancer Research Institute, Tomsk National Research Medical Center, Russian Academy of Sciences, Tomsk, Russia

**Keywords:** multi-omics, transcriptomics, proteomics, valve interstitial cells, calcification, aortic valve

## Abstract

Heart valve calcification is an active cellular and molecular process that partly remains unknown. Osteogenic differentiation of valve interstitial cells (VIC) is a central mechanism in calcific aortic valve disease (CAVD). Studying mechanisms in CAVD progression is clearly needed. In this study, we compared molecular mechanisms of osteogenic differentiation of human VIC isolated from healthy donors or patients with CAVD by RNA-seq transcriptomics in early timepoint (48 h) and by shotgun proteomics at later timepoint (10th day). Bioinformatic analysis revealed genes and pathways involved in the regulation of VIC osteogenic differentiation. We found a high amount of stage-specific differentially expressed genes and good accordance between transcriptomic and proteomic data. Functional annotation of differentially expressed proteins revealed that osteogenic differentiation of VIC involved many signaling cascades such as: PI3K-Akt, MAPK, Ras, TNF signaling pathways. Wnt, FoxO, and HIF-1 signaling pathways were modulated only at the early timepoint and thus probably involved in the commitment of VIC to osteogenic differentiation. We also observed a significant shift of some metabolic pathways in the early stage of VIC osteogenic differentiation. Lentiviral overexpression of one of the most upregulated genes (ZBTB16, PLZF) increased calcification of VIC after osteogenic stimulation. Analysis with qPCR and shotgun proteomics suggested a proosteogenic role of ZBTB16 in the early stages of osteogenic differentiation.

## Introduction

Heart valve calcification is an active cellular and molecular process that mostly remains unknown (1). Calcification of the aortic valve causing aortic stenosis is the most common pathology of heart valves in the Western world. Its occurrence increases exponentially with age (2). The only available treatment for calcific aortic valve disease (CAVD) is open-heart surgery or transcatheter aortic valve replacement (3). Thus, there is an urgent clinical need to develop pharmacological inhibition of calcification. Understanding the cellular and molecular mechanisms of calcification is essential for development of pharmacological inhibition.

Valve interstitial cells (VIC) are located between components of the extracellular matrix inside the valve leaflet (4). This heterogeneous group of cells is considered to play a key role in the pathogenesis of aortic valve stenosis and calcification (5). Under the influence of pathological biochemical and biomechanical stimuli, VIC can differentiate in an osteogenic direction, which eventually leads to aortic valve calcification (6).

One of the emerging approaches to unravel insight into the mechanisms of various diseases is a comprehensive and unbiased assessment of the whole set of proteins or transcripts by specific omics technologies such as proteomics and RNA sequencing (RNAseq). Our study aimed to investigate the differences of proteins, genes, and signaling of human VIC obtained from healthy donors and patients with aortic valve stenosis. Furthermore, to compare the molecular mechanisms of osteogenic differentiation in these groups using a paired multi-omics approach. Another aim was to identify novel players of calcification and identify perspective target(s) for anti-CAVD pharmacotherapy.

## Materials and methods

### Cell cultures

The study protocols were approved by the local Ethics Committee of the Almazov Federal Medical Research Centre and the Regional Ethics Committee South East Norway and performed in accordance with principles of the Declaration of Helsinki. In this study only valves from male donors were included in order to eliminate possible gender differences. All patients gave written informed consent. Human VIC were isolated from tricuspid aortic valves (*n* = 12) explanted during aortic valve replacement due to calcific aortic valve disease (CAVD) at the National Almazov Research Centre, Saint Petersburg, Russia and the Department of Cardiothoracic Surgery, Oslo University Hospital, Oslo, Norway. Patients with known infective endocarditis and rheumatic disease were excluded from the study. VIC from normal aortic valves which were used as a control group were isolated from healthy tricuspid aortic valves obtained from explanted hearts from recipients of heart transplantation (*n* = 12).

To isolate VIC, valve leaflets were washed in PBS and incubated with 0.2% collagenase type 4 solution for 24 h at 37°C. Then the tissue was pipetted repeatedly to break up the tissue mass and centrifuged at 300 *g* for 5 min. The pellet containing VIC were resuspended in basic culture medium consisted of DMEM (Gibco) supplemented with 15% FBS (HyClone, GE Healthcare), 2 mM L-glutamine (Gibco), and 100 units/ml penicillin/streptomycin (Gibco), and plated on T75 flask.

VIC were cultured in standard growth medium (DMEM supplemented with 15% FBS and 100 units/ml penicillin/streptomycin at 37°C in 5% CO_2_ until confluence of 70–80% before passaging. Cells from passages 3–5 were used for all experiments.

### Induction of osteogenic differentiation

To induce osteogenic differentiation of VIC, we used classic osteogenic medium: DMEM supplemented with 10% FBS, 2 mM L-glutamine, 100 units/ml penicillin/streptomycin, 50 mg/ml ascorbic acid, 0.1 mM dexamethasone and 10 mM b–glycerophosphate.

The cells were plated: in 24-well tissue culture plates (33 × 10^3^ cells per well) for Alizarin red staining; in 6-well tissue culture plates (220 × 10^3^ cells per well) for RNA isolation; in 100-mm tissue culture dishes (750 × 10^3^ cells per dish) for lysates obtaining. The cells were seeded in basic culture medium for 24 h at 37°C, 5% CO_2_. The next day osteogenic differentiation was induced. The medium was changed twice a week over a 21-day differentiation period.

### Alizarin red staining

Calcium deposits were visualized by Alizarin Red staining. Cells were washed with PBS, fixed in 70% ethanol for 60 min, washed twice with distilled water and stained using Alizarin Red solution (Sigma).

### qPCR

RNA from cultured cells was isolated using ExtractRNA (Eurogene, Russia) by standard phenol/chloroform extraction procedure in accordance with the manufacturer’s instructions. For RNA isolation, VIC were cultured for 48 h in the presence of osteogenic medium and in standard cultivation conditions. Total RNA (1 μg) was reverse transcribed with MMLV RT kit (Eurogen, Russia). Real-time PCR was performed with 1 μL cDNA and SYBRGreen PCRMastermix (Eurogen, Russia) in the Light Cycler system using specific forward and reverse primers for target genes. Corresponding gene expression level was normalized to GAPDH from the same samples. Changes in target genes expression levels were calculated as fold differences using the comparative ΔΔCT method. All primers sequences can be presented at request.

### RNA-seq transcriptomics

RNA quality was assessed by capillary electrophoresis using an Agilent Bioanalyzer 2100 (Agilent Technologies).

RNA-sequencing was carried out on the equipment of the Core Facility “Medical Genomics” (Tomsk NRMC) and the Tomsk Regional Common Use Center. The technology of highly processive sequencing of libraries prepared from total RNA was used to determine the transcriptomic profile of VIC cultures and evaluate differentially expressed genes, as well as analyze the involved signaling pathways. RNA was isolated from cell cultures from healthy aortic valves (*n* = 6) and from CAVD patients (*n* = 6) using standard phenol-chloroform procedure. Library preparation was performed using the Illumina TruSeq Stranded mRNA kit according to the manufacturer instructions. In brief, on the first library preparation stage, total RNA was incubated with oligo(dT) magnetic particles, while the particle hybridizes the fraction poly-adenylated RNA. After purification of the poly-adenylated fraction, a reverse transcription reaction was performed using a six-membered random primer. On the next step, the second DNA strand was synthesized. The acquired 3’ pre-library DNA was adenylated and a paired end adapter ligation reaction was performed. After ligating the adapter, libraries were amplified using indexed primers. On the next step, the libraries were checked using the instrument Agilent Bioanalizer 2100. Libraries were sequenced on the Illumina NextSeq550 platform using single-ended reagents. All samples were run simultaneously. The raw data obtained by the sequencer was analyzed using software packages STAR, Rsem, DEseq2. For assembly were used GRCH38 reference using Gencode coding region annotation v28. The obtained data on differential expression was visualized by software packages included in the R programming environment and Phantasus program. Involved paths were explored using the base GSEA path data and the corresponding R environment package.

### Shotgun proteomics of VIC under osteogenic differentiation

Mass spectrometry-based proteomic analyses were performed by the Proteomics Core Facility, Department of Biosciences, University of Oslo. This facility is a member of the National Network of Advanced Proteomics Infrastructure (NAPI), which is funded by the Research Council of Norway INFRASTRUKTUR-program (project number: 295910).

Material obtained from patients with aortic valve stenosis (*n* = 8) and healthy donors (*n* = 8) was used for proteomic analysis.

On the 10th day after induction of osteogenic differentiation, the cells were lysed in RIPA buffer (ThermoFisher Scientific) supplemented with protease inhibitors Roche cOmplete™ Protease Inhibitor Cocktail (Sigma-Aldrich, United States). After isolation, the protein concentration was measured, and its quality was checked using PAGE electrophoresis.

The cell slurry was homogenized with a pestle (20x) for mechanical breakage of the cells followed by sonication using an Ultrasonic processor (Vibra-Cell, Sonics and Materials Inc., Newtown, CT, United States). Samples were centrifuged at 16,000 *g* for 20 min at 4°C. To 40 μl, four volumes of ice-cold acetone was added, vortexed and precipitated at −20°C overnight. Samples were centrifuged at 16,000 × *g* for 20 min at 4°C and the supernatant was discarded. Proteins were re-dissolved in 50 μl 6 M urea and 100 mM ammonium bicarbonate, pH 7.8. For reduction and alkylation of cysteines, 2.5 μl of 200 mM DTT in 100 mM Tris–HCl, pH 8 was added and the samples were incubated at 37°C for 1 h followed by addition of 7.5 μl 200 mM iodoacetamide for 1 h at room temperature in the dark. The alkylation reaction was quenched by adding 10 μl 200 mM DTT at 37°C for 1 h. Subsequently, the proteins were digested with 10 μg trypsin for 16 h at 37°C. The digestion was stopped by adding 5 μl 50% formic acid and the generated peptides were purified using an OMIX C18-SPE, 10 μl (Agilent, Santa Clara, CA, United States), and dried using a Speed Vac concentrator (Concentrator Plus, Eppendorf, Hamburg, Germany).

The tryptic peptides were dissolved in 10 μl 0.1% formic acid/2% acetonitrile and 5 μl analyzed using an Ultimate 3000 RSLCnano-UHPLC system connected to a Q Exactive mass spectrometer (Thermo Fisher Scientific, Bremen, Germany) equipped with a nano electrospray ion source. For liquid chromatography separation, an Acclaim PepMap 100 column (C18, 2 μm beads, 100 Å, 75 μm inner diameter, 50 cm length) (Dionex, Sunnyvale CA, United States) was used. A flow rate of 300 nL/min was employed with a solvent gradient of 4–35% B in 100 min, to 50% B in 20 min and then to 80% B in 3 min. Solvent A was 0.1% formic acid and solvent B was 0.1% formic acid/90% acetonitrile. The mass spectrometer was operated in the data-dependent mode to automatically switch between MS and MS/MS acquisition. Survey full scan MS spectra (from m/z 400 to 2,000) were acquired with the resolution *R* = 70,000 at m/z 200, after accumulation to a target of 1e5. The maximum allowed ion accumulation times were 60 ms. The method used allowed sequential isolation of up to the ten most intense ions, depending on signal intensity (intensity threshold 1.7e4), for fragmentation using higher-energy collisional induced dissociation (HCD) at a target value of 1e5 charges, NCE 28, and a resolution *R* = 17,500. Target ions already selected for MS/MS were dynamically excluded for 30 s. The isolation window was m/z = 2 without offset. For accurate mass measurements, the lock mass option was enabled in MS mode. Data were acquired using Xcalibur v2.5.5 and raw files were processed using ProteoWizard release version 3.0.331.

The identification and quantification of proteins from mass spectrometric data was carried out in the MaxQuant software in the “Label-free” quantification mode by default settings. MaxQuant output was analyzed in R by sparse partial least squares discriminant analysis (sPLS-DA) and differential expression analysis by “Limma” package. There were two groups of donors collected in the 2018 and 2019 years which were analyzed independently, but by the same method, in the 2018 and 2019 subsequently. Thus, we excluded batch effect associated with the year according to “Limma” package recommendations.

Functional annotation was performed by the DAVID―Database for Annotation, Visualization and Integrated Discovery (v6.8^1^, accessed on 07/02/2022; and by SIGNAL―Selection by Iterative pathway Group and Network Analysis Looping [v1.0^2^; accessed 07/02/2022; (7)].

The mass spectrometry proteomics data and protein identification results have been deposited to the ProteomeXchange Consortium *via* the PRIDE partner repository.

### Overexpression of ZBTB16 by lentiviral transduction

For overexpression we constructed plasmid for ZBTB16 overexpression by restriction cloning of full sequence of ZBTB16 to pCIG3 (pCMV-IRES-GFP version 3) plasmid purchased from Adgene (Plasmid #78264^3^; accessed 07/02/2022). Full-sized fragment of ZBTB16 mRNA was amplified from TetO-FUW (Addegene^4^) with the targeted insert (ZBTB16) using the primers with extinctions for restriction cloning: F: ATTCTGTAGAATTCGGCCACCATGGATCTGACAAAAAT GGGCAT; R: ATTCTGTAGGATCCTCACACATAGCACAGG TAGAGGT.

Lentiviral production was performed as described previously by three plasmid system. In brief, 100-mm dishes of subconfluent 293T cells were co-transfected with 15 μg pCIG3-ZBTB16, 5.27 μg pMD2.G and 9.73 μg pCMV-dR8.74psPAX2 packaging by polyethylenimine (PEI). The following day, the medium was changed to the fresh one and the cells were incubated for 24 h to obtain high-titer virus production. Produced lentivirus was concentrated from the supernatant by ultracentrifugation, resuspended in 1% BSA/PBS and frozen in aliquots at −80°C. The virus titer was defined by GFP-expressing virus; the efficiency of VIC transduction was 85–90% by GFP.

Lentiviral packaging plasmids were a generous gift from Prof. Didier Trono (École Polytechnique Fédérale de Lausanne, Switzerland). pGa981-6 plasmid was a gift from Prof. Urban Lendahl (Karolinska Institutet, Stockholm, Sweden).

Physiological effect of ZBTB16 overexpression was evaluated by Alizarine red staining and qPCR on *Runx2*, *Col1A1* and *Zbtb16* as described above.

### Proteomics analysis of physiological effect of ZBTB16 overexpression

To evaluate physiological effect of ZBTB16 overexpression, we performed proteomics analysis of VIC in control (transduction with empty vector) and VIC with overexpression of ZBTB16 during osteogenic differentiation. Proteomic analysis was performed by “shotgun” approach, but analysis was performed by tandem mass spectrometry with trapped ion mobility at St. Petersburg State University core facility center “Molecular and Cell technologies.”

On the 10th day after the induction of osteogenic differentiation, the cells were lysed in RIPA buffer (ThermoFisher, United States) supplemented with protease inhibitors Roche cOmplete™ Protease Inhibitor Cocktail (Sigma-Aldrich, United States). The cell slurry was sonicated on Ultrasonic bath with ice for 20 min. Samples were centrifuged at 16,000 *g* for 20 min at 4°C. To 100 μl, four volumes of ice-cold acetone was added, vortexed and precipitated at −20°C overnight. Samples were centrifuged at 16,000 × *g* for 20 min at 4°C and the supernatant was discarded. Protein pellet was washed by acetone, air-dried and re-dissolved in 8M Urea/50 mM ammonium bicarbonate (Sigma Aldrich, United States). Then the protein concentration was measured by QuDye Protein Quantification Kit (Lumiprobe, Russia) in Qubit 4.0 fluorometer (Thermo Fisher, United States).

20 μg of each sample were incubated for 1 h at 37°C with 5 mM DTT (Sigma Aldrich, United States) with subsequent incubation in 15 mM iodoacetamide for 30 min in the dark at RT (Sigma Aldrich, United States). Next, the samples were diluted with seven volumes of 50 mM ammonium bicarbonate and incubated for 16 h at 37°C with 400 ng of Trypsin Gold (ratio 1:50; Promega, United States). The sample was mixed with formic acid (Sigma Aldrich, United States) to 1% final concentration, evaporated in Labconco Centrivap Centrifugal Concentrator and desalted with C18 ZipTip (MilliporeSigma, United States) according to manufacturer recommendations. Desalted peptides were evaporated and dissolved in water/0.1% formic acid for further LC-MS/MS analysis.

Approximate 500 ng of peptides were used for shotgun proteomics analysis by nanoHPLC-MS/MS with trapped ion mobility in TimsToF Pro mass spectrometer (Bruker Daltonics, Germany). HPLC was performed in two-column separation mode with Acclaim PepMap 5 mm Trap Cartridge (Thermo Fisher, United States) and Bruker Fifteen separation column (C18 ReproSil AQ, 150 mm × 0.75 mm, 1.9 μm, 120 A; Bruker Daltonics, Germany) in gradient mode with 400 nL/min flow rate. Phase A was water/0.1% formic acid, phase B was acetonitrile/0.1% formic acid. The gradient was from 2% to 30% phase B for 42 min, then to 95% of phase B for 6 min with subsequent wash with 95% phase B for 6 min. CaptiveSpray ion source was used for electrospray ionization with 1,600 V of capillary voltage, 3 L/min N_2_ flow, and 180°C source temperature. MS/MS acquisition was performed in automatic DDA PASEF mode with 0.5 s cycle in positive polarity with the fragmentation of ions with at least two charges in m/z range from 100 to 1,700 and ion mobility range from 0.85 to 1.30 1/K0.

Protein identification was performed in Peaks Xpro software (Bioinformatics Solutions Inc., Canada) using human protein SwissProt database^5^ (accessed on 07/02/2022; organism: Human [9606]; uploaded on 2 March 2021; 20,394 sequences) and protein contaminants database CRAP^6^ (version of 4 March 2019; accessed on 07/02/2022). The search parameters were: parent mass error tolerance 10 ppm and fragment mass error tolerance 0.05 Da, protein and peptide FDR less than 1%, two possible missed cleavage sites, proteins with at least two unique peptides were included for further analysis. Cysteine carbamidomethylation was set as fixed modification. Methionine oxidation, acetylation of protein N-term, asparagine, and glutamine deamidation were set as variable modifications.

The mass spectrometry proteomics data and protein identification results have been deposited to the ProteomeXchange Consortium *via* the PRIDE partner repository with the dataset identifier PXD031572.

Reviewer account details:

Username: reviewer_pxd031572@ebi.ac.uk

Password: X4dvODZ9

For statistical analysis we used the proteins with NA in less than 85% of samples, then performed imputation of missed values by k-nearest neighbors with subsequent log_2_-transformation and quantile normalization. Finally, we performed analysis of differential expression by ‘‘limma’’ package. Functional annotation was performed by the Database for Annotation, Visualization and Integrated Discovery (DAVID) v6.8^7^ (accessed on 07/02/2022).

## Results

### Global transcriptomic and proteomic signatures differ in VIC from patients with or without CAVD before and after osteogenic differentiation

VIC from healthy (*n* = 6) and calcified aortic valves (*n* = 6) were compared after cultivation in standard conditions and after osteogenic differentiation by transcriptomics (after 48 h) and by proteomics after 10 days.

**FIGURE 1:**
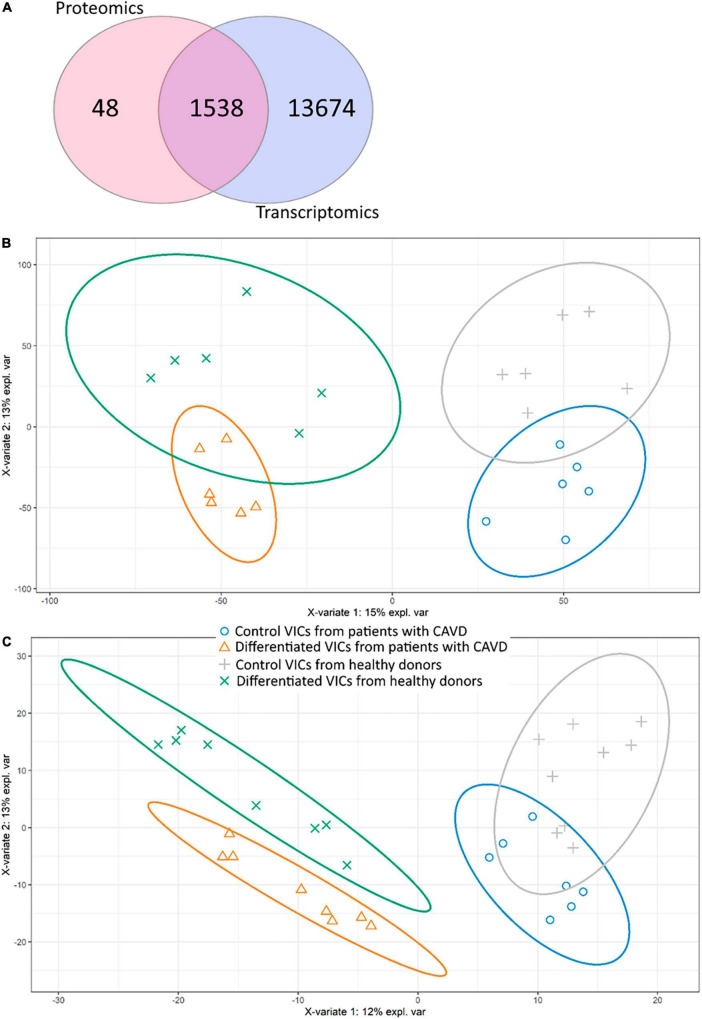
Comparison of proteomic and transcriptomic profiles of human valve interstitial cells (VIC) from patients with or without calcific aortic valve disease (CAVD) in standard cultivation (control) and in osteogenic medium (differentiated). **(A)** Venn diagram shows the number of unique gene products identified by shotgun proteomics and RNA-seq transcriptomics. (b-c) Partial least squares-discriminant analysis (PLS-DA) score plots based on transcriptomic **(B)** and proteomic **(C)** data. Yellow triangles—differentiated VIC from patients with CAVD, green crosses—differentiated VIC from healthy donors, gray pluses—control VIC from healthy donors, blue circles—control VIC from patients with CAVD.

After bioinformatics processing, we identified 17,850 transcripts and 1,594 proteins. Despite different time points, most of the transcripts corresponding to proteins found by proteomics were also identified in RNA-seq (Figure 1A).

We performed analysis on the obtained data by Partial Least Squares discriminant analysis (PLS-DA; Figures 1B,C). Control and differentiated cells formed distinct clusters, while VIC from healthy donors and patients with CAVD demonstrated partly overlapping groups on both proteomics and transcriptomics data. Nevertheless, proteomics data show that VIC from healthy and diseased donors represent distinct clusters on the 10th day after induction of osteogenic differentiation (Figure 1C). Totally four predicted clusters were formed. Furthermore, VIC from patients with CAVD have physiological differences which influence osteogenic differentiation *in vitro*.

However, we were unable to identify transcripts or proteins which were different between VIC from diseased and healthy donors. The only exception was protein arginine *N*-methyltransferase 5 (PRMT5, O14744) which was downregulated in control VIC from patients with CAVD by proteomics data compared to VIC from healthy aortic valves (Log_2_FC = −20.16; adjusted *P*-value < 0.001).

### Molecular mechanisms of osteogenic differentiation in VIC

**FIGURE 2:**
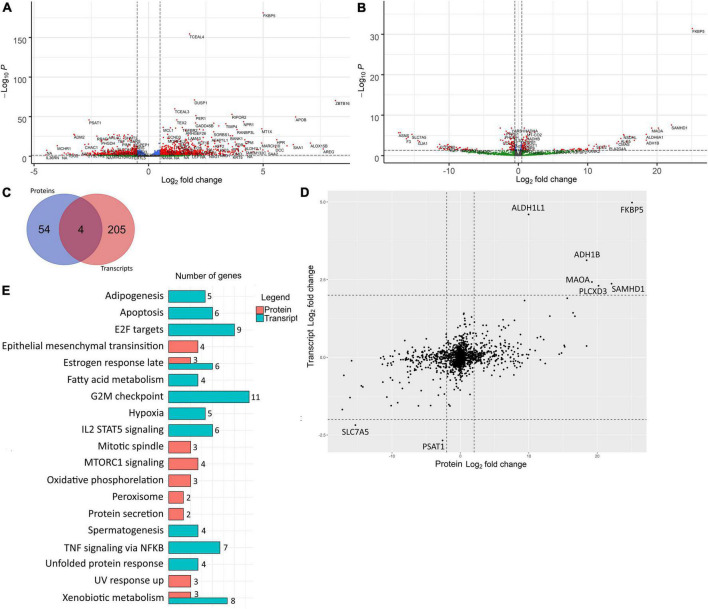
Differentially expressed genes involved in osteogenic differentiation of human valve interstitial cells (VIC) from healthy and calcified valves were identified by transcriptomics and proteomics analysis at early and late time points respectively. **(A)** Volcano plot of RNA-seq analysis of control VIC and 48 h after induction of osteogenic differentiation. Log_2_ fold change—level of change in expression-Log10P—logarithm of *P*-value. Dotted lines cut off transcripts with *P*-value < 0.05 and Log_2_ fold change > |1|. **(B)** Volcano plot of proteomics analysis of control VIC and 10 days after induction of osteogenic differentiation Log_2_ fold change—level of change in expression-Log_10_P—logarithm of *P*-value. Dotted lines cut off transcripts with *P*-value < 0.05 and Log_2_ fold change > |1|. **(C)** Venn diagram representing overlapping of DEGs with Log_2_ fold change > |2| between control and differentiated VIC founded in proteomic and transcriptomic data. **(D)** Scatter plot of comparison of Log_2_Fold Change of genes identified in both proteomic and transcriptomic data. The dotted line separates the border of Log_2_ fold change > |1|. **(E)** Bar chart representing the number of DEGs with Log_2_ fold change > |2| found in proteomic and transcriptomic involved in biological processes based on their overlapping with information in Hallmark gene sets in MSigDB database. All represented overlaps have FDR q-values less than 0.05.

We performed an analysis of differentially expressed genes (DEGs) between control and differentiated VIC. While we quantified products of the same genes on different levels (transcripts and proteins) we translated transcript and protein names to corresponding gene names. Therefore, further we will use DEGs abbreviation for both RNA-seq and shotgun proteomics with specific clarification in which level expression was quantified. Two hundred-and-fourteen and 27 DEGs were up-regulated and 66 and 31 DEGs were down-regulated in transcriptomics and proteomics data, respectively, with at least fourfold difference in the cells after induction of osteogenic differentiation (Figures 2A,B).

These two datasets were from the same donors, but at different timepoints. Therefore, the overall Pearson correlation between proteomic and transcriptomic data was only 0.39. Accordingly, most of the major DEGs were unique for each time point (Figure 2C). Nevertheless, there were no DEGs with opposite expression levels in proteomic and transcriptomic data (Figure 2D). DEGs in early and later time points were involved in different biological processes except for estrogen response and xenobiotic metabolism which were enriched in both proteomic and transcriptomic DEGs (Figure 2E). After 48 h, most of the unique DEGs were associated with the cell cycle (“Apoptosis,” “E2F targets,” “G2M checkpoint”), differentiation (“Adipogenesis”), and signaling cascades (“Il2 stat5 signaling,” “TNF signaling *via* NFKB”). At the later stage, there are DEGs associated with another signaling cascade (“mTORc1 signaling”) and with metabolic changes (“Peroxisome,” “Oxidative phosphorylation,” “Epithelial mesenchymal transition,” “UV response up,” “Protein secretion”).

Similar results were obtained by the enrichment analysis against KEGG database. The transcriptomics data at 48 h showed that the DEGs were involved in the “Cell cycle” (8 DEGs, q-value 2.86 e^–5^), “Drug metabolism cytochrome P450” (7 DEGs, q-value 1.81 e^–5^) “neuroactive ligand receptor interaction” (7 DEGs, q-value 1.74 e^–2^) “Tyrosine metabolism” (4 DEGs, q-value 4.1 e^–3^) “Metabolism of xenobiotics by cytochrome P450” (4 DEGs, q-value 1.74 e^–2^), “Glycine, serine and threonine metabolism” (3 DEGs, q-value 1.74 e^–2^) and “Fatty acid metabolism” (3 DEGs, q-value 3.56 e^–2^). While DEGs found based on proteomics data on the later stage are involved in “Huntington’s disease” (4 DEGs, q-value 1.64 e^–2^) and “Cardiac muscle contraction” (3 DEGs, q-value 1.64 e^–2^).

**FIGURE 3:**
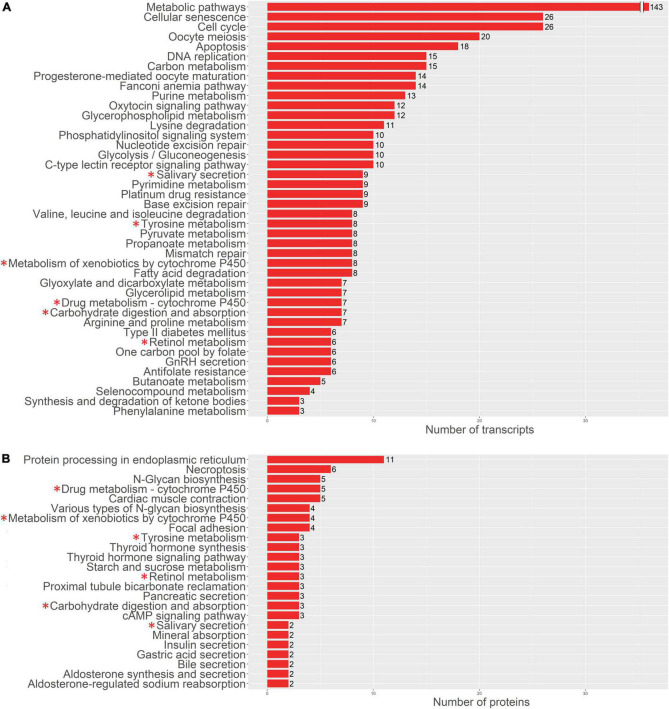
Pathway enrichment analysis of upregulated genes involved in osteogenic differentiation of human aortic valve interstitial cells (VIC) by KEGG: biological processes database. Abscissa axis represents biological processes associated with VIC osteogenic differentiation in early timepoint by RNA-seq transcriptomics data **(A)** or later timepoint by shotgun proteomics data **(B)**; ordinate axis represents number of identified differentially expressed transcripts **(A)** or proteins **(B)** enriched in corresponding biological process. Asterisk marked KEGG (*): biological processes enriched in both proteomics and transcriptomics data.

**FIGURE 4:**
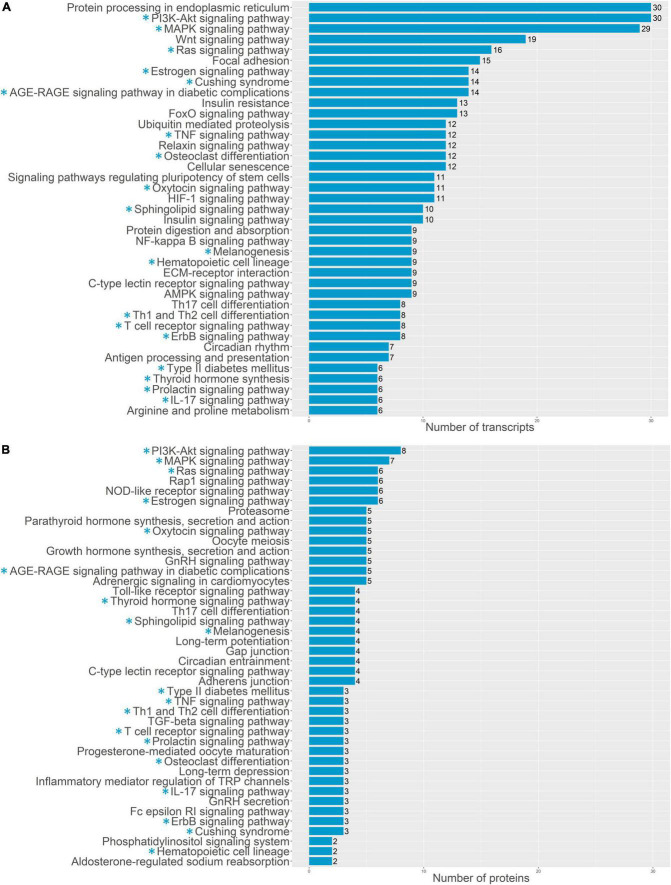
Pathway enrichment analysis of upregulated genes involved in osteogenic differentiation of human aortic valve interstitial cells (VIC) by KEGG: biological processes database. Abscissa axis represents biological processes associated with VIC osteogenic differentiation in early timepoint by RNA-seq transcriptomics data **(A)** or later timepoint by shotgun proteomics data **(B)**; ordinate axis represents number of identified differentially expressed transcripts **(A)** or proteins **(B)** enriched in corresponding biological process. Asterisk marked KEGG (*): biological processes enriched in both proteomics and transcriptomics data.

For a more detailed analysis of enriched biological processes, we performed enrichment analysis of our transcriptomics and proteomics data sets against KEGG: biological processes database *via* signal-net analysis without additional fold change filtration (see footnote 2) [accessed 01.02.2022; (7); Figures 3, 4]. We found a significant shift in VIC physiology in the early stages of osteogenic differentiation associated with the upregulation of many metabolic pathways. Good accordance was observed of proteomics and transcriptomics data at two different time points. Downregulated biological processes were more similar between time points while most of the upregulated biological processes were specific for either early or later timepoint (Figures 3, 4).

**FIGURE 5:**
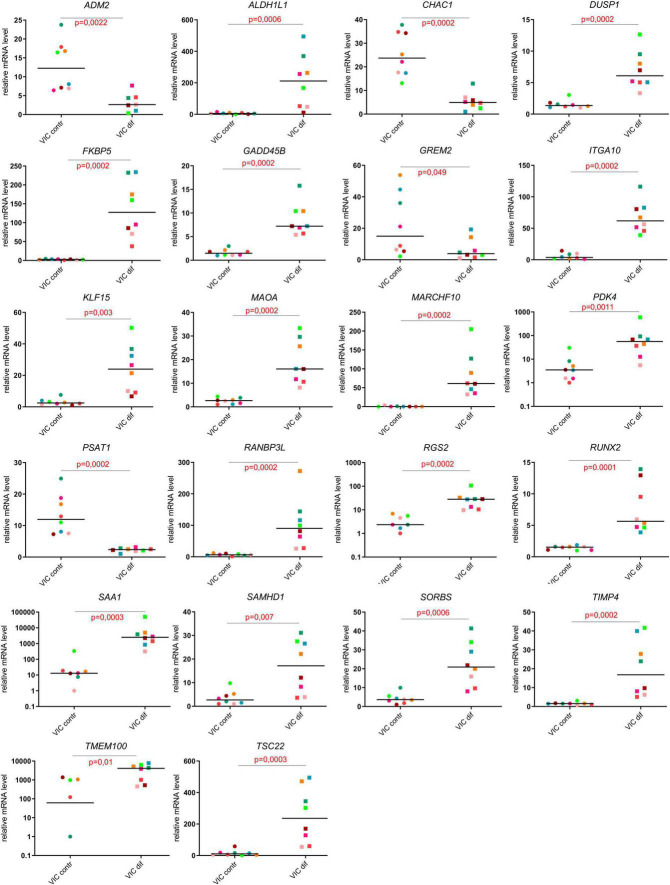
Evaluation of the expression levels of the most responsive genes in comparison of undifferentiated and differentiated human valve interstitial cells (VIC) from healthy donors and patients with CAVD using qPCR.

For a more detailed study of inducers and participants of osteogenic differentiation, we present the top 24 up- or downregulated DEGs ranked according to statistical parameters and *Runx2* as one of the central regulators of osteogenic differentiation (log2Fold Change, *p*-value; Figure 5). The RNA-seq data for all 24 genes were all confirmed by the results of qPCR analysis (Figure 5).

The top upregulated genes included cell cycle regulators and transcriptional factors involved in metabolism (ALDH1L1, DUSP1, ITIH3, MARCH10, PDK4, MARCH10, PDK4, RGS2, SAA1, SAMHD1, TIMP4, TSC22; Figure 5); genes that regulate the processes of differentiation and cell adhesion (FKBP5, ITGA10, KLF15, MAOA, SORBS1, TIMP4; Figure 5). Some of genes associated with Notch and BMP signaling pathways were activated (RANBP3L, TMEM100; Figure 5), while others were suppressed (CHAC1, GREM2; Figure 5) upon induction of VIC osteogenic differentiation. Finally, we noted some genes that mediate cell proliferation and metabolic processes in the regulation of cardiovascular homeostasis (ADM2, PSAT1, RRAD).

### ZBTB16 as an enhancer of osteogenic differentiation

**FIGURE 6:**
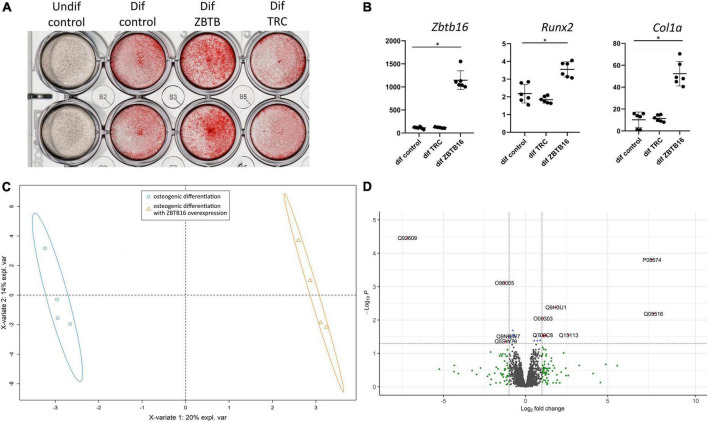
Effect of ZBTB16 (PLZF) on human valve interstitial cells (VIC) osteogenic differentiation. **(A)** Results of Alizarine red stain of VIC cultured in standard medium (undif control), osteogenic medium (dif control), osteogenic medium with transduction by an empty vector (dif TRC), osteogenic medium with transduction by construction for ZBTB16 overexpression (dif ZBTB). **(B)** Results of qPCR of *Zbtb16*, *Runx2*, and *Col1a* in VIC cultured in standard medium (undif control), osteogenic medium (dif control), osteogenic medium with transduction by construction for ZBTB16 overexpression (dif ZBTB). *p-value < 0.05. **(C)** Clusterization by sparse partial least square discriminant analysis (sPLS-DA). **(D)** Volcano plot demonstrating results of analysis of differential expression by limma between VIC in osteogenic conditions and VIC in osteogenic conditions with ZBTB16 overexpression. Log_2_ fold change—level of change in expression -Log_10_P—logarithm of adjusted p–value. Dotted lines cut off transcripts with adjusted *p*-value < 0.05 and Log_2_ fold change > |1|.

One of the most upregulated early proteins involved in osteogenic differentiation of VIC was ZBTB16 (PLZF; Figure 2). To investigate its possible role in osteogenic differentiation, we performed lentiviral transduction by a genetic construction bearing full-length ZBTB16 gene. Overexpression of ZBTB16 (confirmed by qPCR and proteomics data; Figures 6B,D) increased mineralization measured by Alizarin red (Figure 6A) as well as the expression level of *Runx2* (master gene of osteogenic differentiation) and *Col1a* (Figure 6B).

To reveal possible molecular mechanisms by which ZBTB16 enhances osteogenic differentiation of VIC, we compared VIC in osteogenic conditions with and without ZBTB16 overexpression by lentiviral transduction using shotgun proteomics with ion mobility. Overall, 3,865 protein groups were identified, 2,293 of which were used for further analysis by sparse partial least square discriminant analysis (sPLS-DA) and differentially expressed genes (DEGs) analysis. We revealed two distinct clusters of VIC with or without overexpression of ZBTB16 (Figure 6C). Only nine upregulated and four downregulated DEGs had at least a twofold difference (Figure 6D and Table 1). Due to its overexpression, ZBTB16 (Q05516) was the mostly upregulated protein (Figures 6B,D).

**TABLE 1:** Differentially expressed proteins between human valve interstitial cells (VIC) in osteogenic conditions and VIC in osteogenic conditions with ZBTB16 overexpression.

Uniprot Accession	Gene name	Log_2_FC	adj.P.Val
**Upregulated proteins**			
Q05516	ZBTB16	7.8	0.00647
P08574	CYC1	7.7	0.00016
Q15113	PCOLCE	2.6	0.02827
Q9H3U1	UNC45A	1.85	0.00415
P50281	MMP14	1.3	0.03024
Q9BVM4	GGACT	1.2	0.02827
Q92504	SLC39A7	1.1	0.03077
O00303	EIF3F	1.1	0.00897
Q709C8	VPS13C	1.05	0.02827
**Downregulated proteins**			
Q92609	TBC1D5	−7.2	3.46e-05
O96005	CLPTM1	−1.3	0.00077
Q5SW79	CEP170	−1.2	0.04325
Q9NQW7	XPNPEP1	−1	0.03077

Log_2_FC — Log_2_ of fold change in expression, adj.P.Val—adjusted *P*-value.

## Discussion

To investigate molecular mechanisms of experimentally induced aortic valve calcification *in vitro*, we performed proteotranscriptomic analysis of human VIC isolated from healthy and calcified aortic valves. VIC from healthy and diseased patients formed partially overlapping clusters of genes before and after osteogenic differentiation. These clusters demonstrated four distinct biological groups which are associated with molecular mechanisms of osteogenic differentiation. Overexpressing ZBTB16, one of the most upregulated genes from the transcriptomic analysis, increased calcification in VIC after osteogenic stimulation.

Omic studies represent an important tool for assessment of molecular mechanisms underlying biological processes. It is not clear, however, which material is best to use. We used primary VIC *in vitro* culture. This has some obvious disadvantages as the cells *in vitro* may be different from the cells inside the living valve leaflet. Several studies have identified changes in transcripts (8) or proteins (9–13) in the blood of CAVD patients. This approach benefits from the availability of the material and can also identify biomarkers for CAVD. An obvious drawback of this approach is a non-specific footprint of the blood transcriptome with minimal aortic valve transcriptome/proteome contribution.

This problem might be solved by a target analysis of aortic leaflets secretome (14, 15), proteomics (16–24), or transcriptomics (25–27) investigations on the whole human leaflets extracted during surgery. Such data give *in vivo* insight into disease pathogenesis. Nevertheless, it has an important drawback providing the mixed data from all cell subtypes present in the calcified valve leaflet. A recent study by Kossar et al. (28) combined both blood and whole aortic leaflets RNA-sequencing and identified extracellular matrix regulators as possible CAVD markers and targets for therapy. Using several sources (whole leaflet, its secretome, and plasma) for omics analysis in CAVD combined with transcriptomics (29) or proteomics (30) have been reported. Finally, to obtain omics data from the cells responsible for valve calcification (namely VIC), proteomics have been performed on primary rat (31), bovine (30, 32), and human (33–35) VIC cultures from both healthy or calcified valves. Unlike investigations in blood and valve leaflets, employment of primary cell cultures provides possibilities for functional analysis in the naïve and osteoblast-like state leading to calcification. By analyzing human VIC populations transcriptome, Xu et al. (36) identified novel functional interactions among resident VIC subpopulations.

Recently, studies combining proteomics and transcriptomics, called multi-omics, have emerged. In a pioneer study, a comprehensive investigation in whole human valve tissue and cell culture was performed. However, this study only employed VIC from calcified valves (37). The present investigation using VIC from both healthy donors and patients with CAVD might fill some gaps of knowledge.

### Valve interstitial cells from patients with CAVD have properties leading to epigenomic changes

As seen in sPLS-DA plots (Figure 1) and in qPCR verification of omics data (Figure 4), there were high biological variation between the individual donors of VIC. Human cell donor-to-donor heterogeneity is known to be high and should be taken into account (38). Thus, observed high variation is assumed to have biological nature. Such high intragroup variation reduces sensitivity of differential expression analysis. Nonetheless, we identified Protein arginine *N*-methyltransferase 5 (PRMT5, O14744) as markedly downregulated in VIC isolated from patients with CAVD. Protein arginine methyltransferases (PRMTs) are enzymes that catalyze methylation on protein arginine residues. Histones are the main PRMTs target, but they also demethylate many other proteins (39). PRMT5 has been suggested to define an osteogenic context. Kota et al. (40) demonstrated that selective inhibition of PRMT5 in murine bone marrow stromal cells led to increased osteoblast differentiation through blockage of symmetric dimethylation of H3R8 and H4R3 (40). Other epigenetic mechanisms were also previously revealed to be involved in CAVD progression. Theodoris et al. (41) demonstrated that Notch1 haploinsufficiency led to differential H3K27ac at Notch1-bound enhancers (41). The epigenetic changes in VIC may form their osteogenic phenotype and promote calcification (42). Targeted comparison of the epigenome of VIC from patients with CAVD and healthy donors needs to be studied in future studies.

Mesenchymal stem cells (MSC) are often used in studies of osteodifferentiation. Molecular mechanisms of osteogenic differentiation of VIC are assumed to be similar, but not the same as osteogenic differentiation of MSCs and osteoblasts (43, 44). Unique molecular mechanisms of VIC osteogenic differentiation may be possible targets for anti-CAVD therapy. We found that molecular mechanisms of VIC osteogenic differentiation described by us differ from mechanisms of MSC osteogenic differentiation described previously. Comparison of MSC and VIC ostedifferentiation is presented in supplementary data.

### ZBTB16 increased *in vitro* aortic valve calcification

Based on proteomics and transcriptomics data, we detected several proteins which have been less studied in cardiovascular calcification, including ZBTB16 (PLZF). ZBTB16 is a highly conservative zinc-finger transcription factor that might be found in a wide range of species from Nematoda to mammals. It has 9 DNA binding zinc finger domains in C-term. In N-term it has POZ domain involved in protein-protein interactions involved in transcription repression, e.g., by recruiting a histone deacetylase (45, 46). ZBTB16 has a vital role in spermatogenesis (47) and is involved in maintaining cell stemness through regulation of self-renewal and differentiation balance (45). In humans, ZBTB16 has no tissue specificity and might be identified in various tissues from the brain to the pancreas [by the data of Human Protein Atlas^8^, accessed 18.01.2022; (48)].

This transcriptional factor is less studied in osteogenic differentiation, but it might be associated with skeletal development and osteoblast differentiation. ZBTB16 is not normally expressed in MSCs, but starts to express at the early stages of osteoblast differentiation of MSCs (the second day from the start of osteogenic differentiation) (45). ZBTB16 expression in MSCs is controlled by epigenetic mechanisms—loss of H3K27me3 was accompanied by a strong gain of H3K27ac across the whole ZBTB16 locus. Moreover, ZBTB16 targets epigenome by itself—ZBTB16 influencing H3K27 acetylation at osteogenic genes and stimulating their expression (49). Therefore, ZBTB16 siRNA knockdown significantly reduced the expression of genes that were normally induced osteoblast committed of BM-MSCs (49).

In our study, ZBTB16 was one of the most upregulated genes during osteogenic differentiation of VIC. Overexpression of ZBTB16 significantly enhanced both matrix mineralization and RUNX2 and Col1A1 expression in VIC. Overexpression of ZBTB16 caused a relatively small number of DEGs. We did not find any described interactions in STRING database^9^ (accessed 30.01.2022) between ZBTB16 and the DEGs induced by ZBTB16 overexpression.

One of the proteins upregulated by ZBTB16 is UNC45A. There is no systematic evaluation of its role in osteogenic differentiation. It is known that loss-of-function in UNC45A causes bone fragility (50). In the United States patent, US20110263675A1 authors provide UNC45A as one of the targets of miR-27a which has an important role in the shift of MSCs from adipogenic to osteogenic differentiation (51, 52).

eIF-3 is also upregulated by ZBTB16 overexpression. eIF-3 is associated with ribosomes where it recruits other eIF-proteins and forms pre-initiation complex. It was demonstrated that eIF-3 binds to specific mRNA 5’ untranslated region and enhances translation of some mRNA involved in cell cycling, differentiation, and apoptosis (53). ZBTB16 has 5’ untranslated exon (54), so we might assume that eIF-3 is involved in ZBTB16 translation.

### Conclusion

We revealed physiological differences between valve interstitial cells (VIC) isolated from healthy donors before and after osteogenic differentiation which confirm pathological changes in VIC associated with CAVD progression. Signaling pathways might provide targets for anti-CAVD therapy. Finally, upregulation of ZBTB16 (PLZF) in VIC enhanced osteodifferentiation of VIC. This protein may be a target to inhibit soft tissue calcification.

## Data availability statement

The data presented in this study are deposited in the ProteomeXchange Consortium *via* the PRIDE partner repository, accession numbers: PXD032212 and PXD031572.

## Ethics statement

The studies involving human participants were reviewed and approved by Ethics Committee of Almazov National Medical Research Center, ethical permit 12.26/2014. The patients/participants provided their written informed consent to participate in this study.

## Author contributions

JV and AM: conceptualization and writing—review and editing. DS, AZ, AL, NB, OK, BZ, and BT: methodology. AL, DS, and AZ: investigation and data analysis. VU, J-PK, and M-LK: material collection. DS and AL: writing—original draft preparation. AM, JV, AK, and K-OS: supervision. All authors read and approved the final manuscript.

## Footnotes

1
https://david.ncifcrf.gov/

2
https://signal.niaid.nih.gov/

3
https://addgene.org/

4
https://www.addgene.org/61543/

5
https://www.uniprot.org/

6
https://www.thegpm.org/crap/

7
https://david.ncifcrf.gov/

8
https://proteinatlas.org/

9
https://string-db.org/

## References

[1] Zebhi B Lazkani M Bark D Jr. Calcific aortic stenosis-a review on acquired mechanisms of the disease and treatments. *Front Cardiovasc Med.* (2021) 8:734175. 10.3389/fcvm.2021.734175 34604358 PMC8486019

[2] Lamprea-Montealegre JA Otto CM. Health behaviors and calcific aortic valve disease. *J Am Heart Assoc.* (2018) 7:e008385. 10.1161/JAHA.117.008385 29431108 PMC5850268

[3] Head SJ Çelik M Kappetein AP. Mechanical versus bioprosthetic aortic valve replacement. *Eur Heart J.* (2017) 38:2183–91. 10.1093/eurheartj/ehx141 28444168

[4] Butcher JT Mahler GJ Hockaday LA. Aortic valve disease and treatment: the need for naturally engineered solutions. *Adv Drug Deliv Rev.* (2011) 63:242–68. 10.1016/j.addr.2011.01.008 21281685

[5] Rutkovskiy A Malashicheva A Sullivan G Bogdanova M Kostareva A Stensløkken K-O Valve interstitial cells: the key to understanding the pathophysiology of heart valve calcification. *J Am Heart Assoc.* (2017) 6:e006339. 10.1161/JAHA.117.006339 28912209 PMC5634284

[6] Dweck MR Boon NA Newby DE. Calcific aortic stenosis: a disease of the valve and the myocardium. *J Am College Cardiol.* (2012) 60:1854–63. 10.1016/j.jacc.2012.02.093 23062541

[7] Katz S Song J Webb KP Lounsbury NW Bryant CE Fraser IDC. Signal: a web-based iterative analysis platform integrating pathway and network approaches optimizes hit selection from genome-scale assays. *Cell Syst.* (2021) 12:338–52.e5. 10.1016/j.cels.2021.03.001 33894945 PMC7613048

[8] Thériault S Gaudreault N Lamontagne M Rosa M Boulanger M-C Messika-Zeitoun D A transcriptome-wide association study identifies PALMD as a susceptibility gene for calcific aortic valve stenosis. *Nat Commun.* (2018) 9:988. 10.1038/s41467-018-03260-6 29511167 PMC5840407

[9] Gil-Dones F Darde VM Alonso-Orgaz S Lopez-Almodovar LF Mourino-Alvarez L Padial LR Inside human aortic stenosis: a proteomic analysis of plasma. *J Proteomics.* (2012) 75:1639–53. 10.1016/j.jprot.2011.11.036 22178735

[10] Satoh K Yamada K Maniwa T Oda T Matsumoto K. Monitoring of serial presurgical and postsurgical changes in the serum proteome in a series of patients with calcific aortic stenosis. *Dis Mark.* (2015) 2015:694120. 10.1155/2015/694120 26078484 PMC4452854

[11] Mourino-Alvarez L Baldan-Martin M Gonzalez-Calero L Martinez-Laborde C Sastre-Oliva T Moreno-Luna R Patients with calcific aortic stenosis exhibit systemic molecular evidence of ischemia, enhanced coagulation, oxidative stress and impaired cholesterol transport. *Int J Cardiol.* (2016) 225:99–106. 10.1016/j.ijcard.2016.09.089 27716559

[12] Olkowicz M Debski J Jablonska P Dadlez M Smolenski RT. Application of a new procedure for liquid chromatography/mass spectrometry profiling of plasma amino acid-related metabolites and untargeted shotgun proteomics to identify mechanisms and biomarkers of calcific aortic stenosis. *J Chromatogr A.* (2017) 1517:66–78. 10.1016/j.chroma.2017.08.024 28851525

[13] Ljungberg J Janiec M Bergdahl IA Holmgren A Hultdin J Johansson B Proteomic biomarkers for incident aortic stenosis requiring valvular replacement. *Circulation.* (2018) 138:590–9. 10.1161/CIRCULATIONAHA.117.030414 29487139

[14] de la Cuesta F Alvarez-Llamas G Gil-Dones F Darde VM Calvo E Lopez JA Secretome of human aortic valves. Methods. *Mol Biol.* (2013) 1005:237–43. 10.1007/978-1-62703-386-2_19 23606262

[15] Alvarez-Llamas G Martin-Rojas T de la Cuesta F Calvo E Gil-Dones F Darde VM Modification of the secretion pattern of proteases, inflammatory mediators, and extracellular matrix proteins by human aortic valve is key in severe aortic stenosis. *Mol Cell Proteomics.* (2013) 12:2426–39. 10.1074/mcp.M113.027425 23704777 PMC3769321

[16] Weisell J Ohukainen P Napankangas J Ohlmeier S Bergmann U Peltonen T Heat shock protein 90 is downregulated in calcific aortic valve disease. *BMC Cardiovasc Disord.* (2019) 19:306. 10.1186/s12872-019-01294-2 31856737 PMC6923932

[17] Martin-Rojas T Mourino-Alvarez L Alonso-Orgaz S Rosello-Lleti E Calvo E Lopez-Almodovar LF iTRAQ proteomic analysis of extracellular matrix remodeling in aortic valve disease. *Sci Rep.* (2015) 5:17290. 10.1038/srep17290 26620461 PMC4664895

[18] Gil-Dones F Martin-Rojas T Lopez-Almodovar LF de la Cuesta F Darde VM Alvarez-Llamas G Valvular aortic stenosis: a proteomic insight. *Clin Med Insights Cardiol.* (2010) 4:1–7. 10.4137/CMC.S3884 20567634 PMC2884338

[19] Matsumoto K Satoh K Maniwa T Araki A Maruyama R Oda T. Noticeable decreased expression of tenascin-X in calcific aortic valves. *Connect Tissue Res.* (2012) 53:460–8. 10.3109/03008207.2012.702818 22827484

[20] Suzuki H Chikada M Yokoyama MK Kurokawa MS Ando T Furukawa H Aberrant glycosylation of lumican in aortic valve stenosis revealed by proteomic analysis. *Int Heart J.* (2016) 57:104–11. 10.1536/ihj.15-252 26742884

[21] Bouchareb R Guauque-Olarte S Snider J Zaminski D Anyanwu A Stelzer P Proteomic architecture of valvular extracellular matrix: FNDC1 and MXRA5 are new biomarkers of aortic stenosis. *JACC Basic Transl Sci.* (2021) 6:25–39. 10.1016/j.jacbts.2020.11.008 33532664 PMC7838057

[22] Lim J Aguilan JT Sellers RS Nagajyothi F Weiss LM Angeletti RH Lipid mass spectrometry imaging and proteomic analysis of severe aortic stenosis. *J Mol Histol.* (2020) 51:559–71. 10.1007/s10735-020-09905-5 32794037 PMC7672660

[23] Schlotter F de Freitas RCC Rogers MA Blaser MC Wu PJ Higashi H ApoC-III is a novel inducer of calcification in human aortic valves. *J Biol Chem.* (2021) 296:100193. 10.1074/jbc.RA120.015700 33334888 PMC7948477

[24] Han RI Hu CW Loose DS Yang L Li L Connell JP Differential proteome profile, biological pathways, and network relationships of osteogenic proteins in calcified human aortic valves. *Heart Vessels.* (2021) 37:347–58. 10.1007/s00380-021-01975-z 34727208 PMC10960607

[25] Padang R Bagnall RD Tsoutsman T Bannon PG Semsarian C. Comparative transcriptome profiling in human bicuspid aortic valve disease using RNA sequencing. *Physiol Genom.* (2015) 47:75–87. 10.1152/physiolgenomics.00115.2014 25547111

[26] Wang J Wang Y Gu W Ni B Sun H Yu T Comparative transcriptome analysis reveals substantial tissue specificity in human aortic valve. *Evol Bioinform Online.* (2016) 12:175–84. 10.4137/EBO.S37594 27493474 PMC4968975

[27] Guauque-Olarte S Droit A Tremblay-Marchand J Gaudreault N Kalavrouziotis D Dagenais F RNA expression profile of calcified bicuspid, tricuspid, and normal human aortic valves by RNA sequencing. *Physiol Genom.* (2016) 48:749–61. 10.1152/physiolgenomics.00041.2016 27495158 PMC6195654

[28] Kossar AP Anselmo W Grau JB Liu Y Small A Carter SL Circulating and tissue matricellular RNA and protein expression in calcific aortic valve disease. *Physiol Genom.* (2020) 52:191–9. 10.1152/physiolgenomics.00104.2019 32089075 PMC7191425

[29] MacGrogan D Martínez-Poveda B Desvignes JP Fernandez-Friera L Gomez MJ Gil Vilariño E Identification of a peripheral blood gene signature predicting aortic valve calcification. *Physiol Genom.* (2020) 52:563–74. 10.1152/physiolgenomics.00034.2020 33044885

[30] Renato M Bertacco E Franchin C Arrigoni G Rattazzi M. Proteomic analysis of interstitial aortic valve cells acquiring a pro-calcific profile. *Methods Mol Biol.* (2013) 1005:95–107. 10.1007/978-1-62703-386-2_8 23606251

[31] Cui L Rashdan NA Zhu D Milne EM Ajuh P Milne G End stage renal disease-induced hypercalcemia may promote aortic valve calcification *via* annexin VI enrichment of valve interstitial cell derived-matrix vesicles. *J Cell Physiol.* (2017) 232:2985–95. 10.1002/jcp.25935 28369848 PMC5575563

[32] Bertacco E Millioni R Arrigoni G Faggin E Iop L Puato M Proteomic analysis of clonal interstitial aortic valve cells acquiring a pro-calcific profile. *J Proteome Res.* (2010) 9:5913–21. 10.1021/pr100682g 20825172

[33] Goto S Rogers MA Blaser MC Higashi H Lee LH Schlotter F Standardization of human calcific aortic valve disease *in vitro* modeling reveals passage-dependent calcification. *Front Cardiovasc Med.* (2019) 6:49. 10.3389/fcvm.2019.00049 31041314 PMC6476921

[34] Yu B Khan K Hamid Q Mardini A Siddique A Aguilar-Gonzalez LP Pathological significance of lipoprotein(a) in aortic valve stenosis. *Atherosclerosis.* (2018) 272:168–74. 10.1016/j.atherosclerosis.2018.03.025 29614432

[35] Khan K Yu B Kiwan C Shalal Y Filimon S Cipro M The role of Wnt/β-catenin pathway mediators in aortic valve stenosis. *Front Cell Dev Biol.* (2020) 8:862. 10.3389/fcell.2020.00862 33015048 PMC7513845

[36] Xu K Xie S Huang Y Zhou T Liu M Zhu P Cell-type transcriptome atlas of human aortic valves reveal cell heterogeneity and endothelial to mesenchymal transition involved in calcific aortic valve disease. *Arterioscler Thromb Vasc Biol.* (2020) 40:2910–21. 10.1161/ATVBAHA.120.314789 33086873

[37] Schlotter F Halu A Goto S Blaser MC Body SC Lee LH Spatiotemporal multi-omics mapping generates a molecular atlas of the aortic valve and reveals networks driving disease. *Circulation.* (2018) 138:377–93. 10.1161/CIRCULATIONAHA.117.032291 29588317 PMC6160370

[38] Phinney DG. Functional heterogeneity of mesenchymal stem cells: implications for cell therapy. *J Cell Biochem.* (2012) 113:2806–12. 10.1002/jcb.24166 22511358

[39] Couto ESA Wu CY Citadin CT Clemons GA Possoit HE Grames MS Protein arginine methyltransferases in cardiovascular and neuronal function. *Mol Neurobiol.* (2020) 57:1716–32. 10.1007/s12035-019-01850-z 31823198 PMC7062579

[40] Kota SK Roening C Patel N Kota SB Baron R. PRMT5 inhibition promotes osteogenic differentiation of mesenchymal stromal cells and represses basal interferon stimulated gene expression. *Bone.* (2018) 117:37–46. 10.1016/j.bone.2018.08.025 30189247 PMC6317875

[41] Theodoris CV Li M White MP Liu L He D Pollard KS Human disease modeling reveals integrated transcriptional and epigenetic mechanisms of NOTCH1 haploinsufficiency. *Cell.* (2015) 160:1072–86. 10.1016/j.cell.2015.02.035 25768904 PMC4359747

[42] Bogdanova M Zabirnyk A Malashicheva A Enayati KZ Karlsen TA Kaljusto ML Interstitial cells in calcified aortic valves have reduced differentiation potential and stem cell-like properties. *Sci Rep.* (2019) 9:12934. 10.1038/s41598-019-49016-0 31506459 PMC6736931

[43] Kostina A Lobov A Semenova D Kiselev A Klausen P Malashicheva A. Context-specific osteogenic potential of mesenchymal stem cells. *Biomedicines.* (2021) 9:673. 10.3390/biomedicines9060673 34204737 PMC8231580

[44] Monzack EL Masters KS. Can valvular interstitial cells become true osteoblasts? A side-by-side comparison. *J Heart Valve Dis.* (2011) 20:449–63. 21863660 PMC3285463

[45] Liu TM Lee EH Lim B Shyh-Chang N. Concise review: balancing stem cell self-renewal and differentiation with PLZF. *Stem Cells.* (2016) 34:277–87. 10.1002/stem.2270 26676652

[46] David G Alland L Hong SH Wong CW DePinho RA Dejean A. Histone deacetylase associated with mSin3A mediates repression by the acute promyelocytic leukemia-associated PLZF protein. *Oncogene.* (1998) 16:2549–56. 10.1038/sj.onc.1202043 9627120

[47] Costoya JA Hobbs RM Barna M Cattoretti G Manova K Sukhwani M Essential role of Plzf in maintenance of spermatogonial stem cells. *Nat Genet.* (2004) 36:653–9. 10.1038/ng1367 15156143

[48] Uhlén M Fagerberg L Hallström BM Lindskog C Oksvold P Mardinoglu A Proteomics. Tissue-based map of the human proteome. *Science.* (2015) 347:1260419. 10.1126/science.1260419 25613900

[49] Agrawal Singh S Lerdrup M Gomes AR van de Werken HJ Vilstrup Johansen J Andersson R Plzf targets developmental enhancers for activation during osteogenic differentiation of human mesenchymal stem cells. *Elife.* (2019) 8:e40364. 10.7554/eLife.40364 30672466 PMC6344081

[50] Esteve C Francescatto L Tan PL Bourchany A De Leusse C Marinier E Loss-of-function mutations in UNC45A cause a syndrome associating cholestasis, diarrhea, impaired hearing, and bone fragility. *Am J Hum Genet.* (2018) 102:364–74. 10.1016/j.ajhg.2018.01.009 29429573 PMC5985364

[51] Federov Y Leake D. Methods of modulating mesenchymal stem cell differentiation. Patent United States US20110263675A1. (2011).

[52] You L Pan L Chen L Gu W Chen J. MiR-27a is essential for the shift from osteogenic differentiation to adipogenic differentiation of mesenchymal stem cells in postmenopausal osteoporosis. *Cell Physiol Biochem.* (2016) 39:253–65. 10.1159/000445621 27337099

[53] Lee AS Kranzusch PJ Cate JH. eIF3 targets cell-proliferation messenger RNAs for translational activation or repression. *Nature.* (2015) 522:111–4. 10.1038/nature14267 25849773 PMC4603833

[54] van Schothorst EM Prins DE Baysal BE Beekman M Licht JD Waxman S Genomic structure of the human Plzf gene. *Gene.* (1999) 236:21–4. 10.1016/S0378-1119(99)00277-2 10433962

